# Influence of Ketotifen, Cromolyn Sodium, and Compound 48/80 on the survival rates after intestinal ischemia reperfusion injury in rats

**DOI:** 10.1186/1471-230X-8-42

**Published:** 2008-09-22

**Authors:** Hei Zi-qing, Gan Xiao-liang, Huang Pin-jie, Wei Jing, Shen Ning, Gao Wan-ling

**Affiliations:** 1 Department of Anesthesiology, The Third Affiliated Hospital, Sun Yat-sen University, Guangzhou, PR China

## Abstract

**Background:**

Mast cells were associated with intestinal ischemia-reperfusion injury, the study was to observe the influence of Ketotifen, Cromolyn Sdium(CS), and Compound 48/80(CP) on the survival rates on the third day after intestinal ischemia-reperfusion injury in rats.

**Methods:**

120 healthy Sprague-Dawley rats were randomly divided into 5 groups, Sham-operated group (group S), model group (group M), group K, group C and group CP. Intestinal damage was triggered by clamping the superior mesenteric artery for 75 minutes, group K, C, and CP were treated with kotifen 1 mg·kg^-1^, CS 50 mg·kg^-1^, and CP 0.75 mg·kg^-1 ^i.v. at 5 min before reperfusion and once daily for three days following reperfusion respectively. Survival rate in each group was recorded during the three days after reperfusion. All the surviving rats were killed for determining the concentration of serum glutamic-oxaloacetic transaminase(AST), glutamic pyruvic transaminase(ALT), the ratio of AST compare ALT(S/L), total protein(TP), albumin(ALB), globulin(GLB), the ratio of ALB compare GLB(A/G), phosphocreatine kinase(CK), lactate dehydrogenase(LDH), urea nitrogen(BUN) and creatinine(CRE) at the 3^rd ^day after reperfusion. And ultrastructure of IMMC, Chiu's score, lung histology, IMMC counts, the levels of TNF-α, IL-1β, IL-6 and IL-10 of the small intestine were detected at the same time.

**Results:**

Intestinal ischemia-reperfusion injury reduced the survival rate. The concentrations of TP, ALB and level of IL-10 in intestine in group M decreased significantly while the concentrations of S/L, LDH and the levels of IL-6 and TNF-α in intestine increased significantly compared with group S (*P *< 0.05). Treatment with Ketotifen and CS increased the survival rate compared with group M (*P *< 0.05), attenuated the down-regulation or up-regulation of the above index (*P *< 0.05). Treatment with CP decreased the survival rate on the 3^rd ^day after reperfusion compared with group M(*P *< 0.05). Group K and C had better morphology in IMMC in the small intestine and in the lungs than in group M and CP, although the Chiu's score and IMMC counts remained the same in the five groups(*P *> 0.05).

**Conclusion:**

Mast cell inhibition after ischemia prior to reperfusion and following reperfusion may decrease the multi-organ injury induced by intestine ischemia reperfusion, and increase the survival rates.

## Background

Intestinal ischemia-reperfusion injury (IIRI) contributes to the pathophysiology of many conditions, including abdominal aortic aneurysm surgery, small bowel transplantation, cardiopulmonary bypass, strangulated hernias, and neonatal necrotizing enterocolitis [1,2]. In addition to localized tissue damages, IIRI induces inflammatory damages in remote organs, particularly in the lung and liver, and is associated with a high mortality [3].

Intestinal mucosal mast cells (IMMC) are particularly frequent in close proximity to epithelial surfaces, where they are strategically located for optimal interaction with the environment and for their putative functions for host defense [4]. Previous studies demonstrated that the de-granulation of IMMC can be induced by oxidants generated in the post-ischemic gut, and the released inflammatory mediators such as histamine and tumor necrosis factor-α(TNF-α) could aggravate the injury to intestine after reperfusion [5,6].

Cordeiro and colleagues reported that administration of diphenhydramine (50 mg/kg, H2 blocker) before reperfusion can significantly reduce the extent of flap necrosis and the neutrophil and mast cell counts caused by ischemia/reperfusion [7]. Kalia found that all ketotifen-pretreated animals (1 mg/kg orally twice a day for 3 days before ischemia) survived in 12 hours after ischemia-reperfusion(I/R), while the ten untreated animals subjected to intestinal I/R failed to survive the reperfusion period (Each group had 12 animals) [8]. The above results prove that administration of antihistaminic agents could decrease IIRI.

The influence of administration of mast cell membrane stabilizer, H2 blocker or mast cell degranulator on the damage and the survival rate caused by IIRI after intestinal ischemia have not previously been well investigated. The purpose of our present investigation was to observe the influence of Ketotifen, cromolyn sodium, and compound 48/80 on the survival after intestinal ischemia-reperfusion injury in rats, and provided a new therapeutic method to treat IIRI.

## Methods

### Establish the small intestinal ischemia-reperfusion injury model in rats and the experimental design

This study proceeded after being reviewed and approved by the Institutional Animal Care and Use Committee in accordance with the ethical principles provided by the Experimental Animal Laboratory of School of Medicine, SUN Yat-sen University. Forty-eight healthy Sprague-Dawley rats (weighing 200–250 g) were randomly divided into four groups. Each of which contained 12 rats, the group I (the ischemia time was 60 min), group II (the ischemia time was 75 min), group III (the ischemia time was 90 min), and group IV (the ischemia time was 120 min). Laboratory temperature was kept at 25–27°C. Surgery was conducted under general anesthesia with intra-peritoneal sodium pentobarbital (45 mg/kg) after they had been fasted for 18 h. The rats' abdomens were opened and their superior mesenteric artery (SMA) were found and clamped for 60, 75, 90 and 120 min respectively. Then the clamp was released and abdominal membrane, muscle and skin were sutured gradually. In addition, 5% Cefoperazone was injected intra-peritoneal to avoid wound infection. Animals were housed individually in wire-bottomed cages, free to eat water and food. The survival rates in each group were observed during the 1^st ^day to the 7^th ^day after intestine ischemia/reperfusion.

Based on the above result, One hundred and twenty healthy Sprague-Dawley rats (200–250 g) were randomly divided into five groups. Each of which contained 24 rats, Sham-operated group(group S), model group(group M), Ketotifen treated group(group K), cromolyn sodium treated group(group C) and compound 48/80 treated group(group CP). Intestinal damages were induced by clamping the superior mesenteric artery for 75 minutes based on the above study. Group K, C, and CP were treated with ketotifen (Sigma; USA) 1 mg·kg^-1^, CS (ICN; USA)50 mg·kg^-1^, and CP (Sigma; USA) 0.75 mg·kg^-1 ^via caudal vein at 5 min before reperfusion, respectively, while group S and M were treated with the same volume of saline. Then the clamp was released and abdominal membrane, muscle and skin were sutured gradually. In addition, 5% Cefoperazone was injected intra-peritoneal to avoid wound infection. Animals were housed individually in wire-bottomed cages, free to eat water and food. The surviving rats in group K, C, and CP were treated with ketotifen 1 mg·kg^-1^, CS 50 mg·kg^-1^, and CP 0.75 mg·kg^-1 ^via caudal vein once daily for 3 days after reperfusion respectively, while group S and M were treated with the same volume saline.

### Survival rates

The survival rates in each group were observed during the 1^st ^day to the 3^rd ^day after intestine ischemia/reperfusion. The state, action, drinking and eating of each surviving rat was also recorded.

### Preparation of specimens and measurements

The surviving rats were sacrificed by anesthetic overdose. 8 rats in each group except only 3 rats in group CP were paunched rapidly on the 3^rd ^day after reperfusion. 2 mL blood was obtained from the inferior vena cava, frozen at -20°C for 5 minutes and centrifuged for 15 minutes at 4,000 r/min. Supernatants were transferred into fresh tubes for evaluation of concentration of glutamic-oxaloacetic transaminase (AST), glutamic pyruvic transaminase (ALT), the ratio of AST compare ALT (S/L), total protein (TP), albumin (ALB), globulin (GLB), the ratio of ALB compare GLB (A/G), phosphocreatine kinase (CK), lactate dehydrogenase (LDH), urea nitrogen (BUN) and creatinine (CRE) through automatic biochemistry analyzer(abbott, USA).

### Intestine histology

A 0.5–1.0 cm intestinal segment was cut 5 cm from the terminal ileum and fixed in 4% formaldehydum polymerisatum, then embedded in paraffin for sectioning. The segment was then stained with hematoxylin-eosin. Damages of intestinal mucosa were evaluated by two different histopathologist according to the criteria of Chiu's method [9]. Criteria of Chiu grading system consists of 5 subdivisions according to the changes of villus and gland of intestinal mucosa: grade 0, normal mucosa; grade 1, development of subepithelial Gruenhagen's space at the tip of villus; grade 2, extension of the space with moderate epithelial lifting; grade 3, massive epithelial lifting with a few denuded villi; grade 4, denuded villi with exposed capillaries; and grade 5, disintegration of the lamina propria, ulceration and hemorrhage.

### Transmission Electron Microscopy

Another 0.5 cm intestinal segment cut 5 cm from the terminal ileum were immersed and fixed in 2.5% glutaraldehyde overnight at 4°C and washed three times in PBS. They were then postfixed in aqueous 1% OsO_4 _and 1% K_3_Fe (CN)_6 _for 1 hour. Following three times of PBS washing, the tissue was dehydrated through a graded series of 30 to 100% ethanol and 100% propylene oxide and immersed in 1:1 mixture of propylene oxide and Polybed 812 epoxy resin for 1 hour. The infiltration solution was then changed to 100% resin. After 24 hours of infiltration, the tissue was embedded in molds and cured at 37°C overnight, followed by additional hardening at 65°C for 2 days. Ultrathin (70 nm) sections were collected on 200-mesh copper grids and stained with 2% uranyl acetate in 50% methanol for 10 minutes, followed by 1% lead citrate for 7 minutes. Sections were photographed using a Hitachi H-600 transmission electron microscope (TOSHIBA, Japan) at 80 kV onto electron microscope film.

### Lung histology

A median sternotomy was performed. The harvested right middle lobe of the lung was fixed in 4% formaldehydum polymerisatum. Paraffin-embedded sections (5 μm) were stained with hematoxylin-eosin and evaluated blindly by two different histopathologist.

### Detection of concentration of protein in intestine

Another segment of 10 cm intestine was cut 5 cm from terminal ileum. The small intestine was washed with frozen saline and dried with suction paper at 4°C. Intestinal tissues were homogenized with normal saline. Intestinal protein (content) was quantified by the Bradford method with a BSA standard kit, provided by Shenerg Biocolor BioScience & Technolgy Company, Shanghai, China.

### Detection of the levels of TNF-α, IL-1β, IL-6 and IL-10 in the intestine

Intestinal tissues were homogenized with normal saline, frozen at -20°C for 5 minutes and centrifuged for 15 minutes at 4,000 r/min. Supernatants were transferred into fresh tubes. The levels of TNF-α, IL-1β, IL-6 and IL-10 were measured using a bead-based immunofluorescence assay (Luminex Inc. Austin, TX, USA)using multiplex cytokine reagents supplied by Linco International, USA. Briefly, antibody-coupled beads were incubated with the samples (antigen), followed by incubation with biotinylated detection antibody and streptavidin-phycoerythrin, respectively. A broad sensitivity range of standards (Linco International), ranging between 1.95 and 32 000 pg/ml were used to help enable the quantization of a dynamic wide range of cytokine concentrations and provide the greatest sensitivity. This captured bead-complexes were then read by the Luminex fluorescent bead-based technology Luminex™ 200 Liquid Array (Luminex Corporation Austin, TX, USA) with a flow-based dual laser detector with real-time digital signal processing to facilitate the analysis of up to 100 different families of colour-coded polystyrene beads and allow multiple measurements of the sample ensuring in the effective quantification of cytokines. The levels of TNF-α, IL-1β, IL-6 and IL-10 in the intestine were indicated as picogram per milligram of protein.

### Immunohistochemical detection of tryptase in intestine

Five-micron-thick sections were prepared from paraffin-embedded intestinal tissues. After deparaffinization, endogenous peroxidase was quenched with 3% H_2_O_2 _in deionised water for 10 minutes. Nonspecific binding sites were blocked by incubating the sections in 10% normal rabbit serum for 1 hour. The sections were then incubated with polyclonal rat anti-mast cell tryptase (dilution 1: 50) for 30 minutes at 37°C, followed by incubating with biotinylated goat-anti-rat IgG at room temperature for 10–15 minutes. After 3 times rinsing of the sample with PBS for 5 minutes, the horseradish-peroxidase-conjugated streptavidin solution was added and incubated at room temperature for 10–15 minutes. The antibody binding sites were visualized by incubating with diaminobenzidine-H_2_O_2 _solution. Sections incubated with PBS instead of the primary antibody were used as negative controls. Brownish granules in the cytoplasm were recognized as positive staining for tryptase. We calculated the tryptase positive mast cells in 5 representative areas at 400× magnification by Image-Pro Plus 5.0 (USA)

### Statistics

Data were expressed as mean ± SD. Analysis of variance was performed using SPSS 11.0 software. One-way analysis of variance was used for multiple comparison. Bonferroni test was used for intra-group comparison or Tamhane's T2 test was used if equal variances was not assumed. Chi-Square test was used to determine the significance of differences of the survival rates. Differences were considered significant when *P *value was less than 0.05.

## Results

### Changes of survival rate after different ischemia time

**Figure 1:**
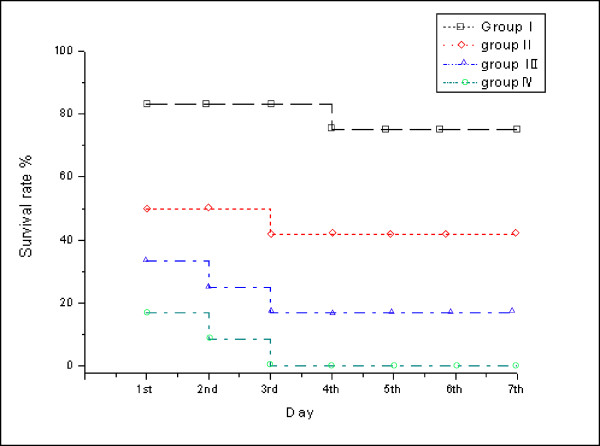
**Changes in survival rate after ischemia and reperfusion injury (establish animal model).** Group I (60 minutes of ischemia), group II (75 minutes of ischemia), group III (90 minutes of ischemia), and group IV (120 mins minute of ischemia).

The 1^st ^to 7^th ^day's survival rates after 60 minutes' intestine ischemia were from 83.3% to 75%. The 1^st ^to the 7^th ^day's survival rates after 75 minutes' intestine ischemia were from 50% to 41.6%. The 1^st ^to 7^th ^day's survival rates after 90 minutes' intestine ischemia were from 33.3% to 16.7%, while the 1^st ^to 7^th ^day's survival rates after 120 minutes' intestine ischemia were from 16.7% to zero. The survival rates on the 3^rd ^day groups I, II, III and group IV were 83.3%, 41.6%, 25%, and 0, respectively. There were almost no animals died in the 4^th ^to the 7^th ^day after reperfusion. (Fig. 1)

### Changes in survival rates and states after operation

The surviving rats in group S recovered more vigorously and vitally on the 3^rd ^day after 75 min intestine ischemia. The survival states of the rats in group M, K and C also recovered vigorously and vitally while those in group CP recovered less vigorously and vitally.

**Figure 2:**
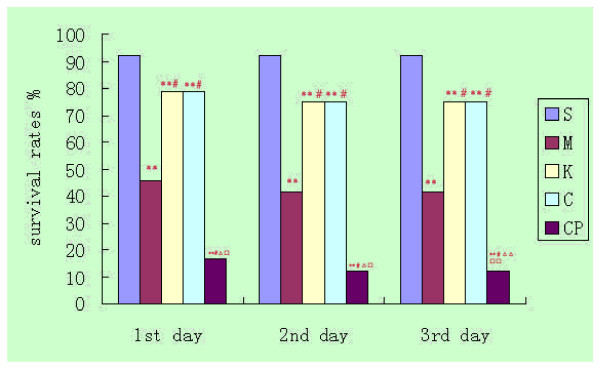
**Changes in the survival rate following 75 min intestine ischemia.** Compared with group S: *P < 0.05,**P < 0.01; compared with group M: ^#^*P *< 0.05, ^##^*P *< 0.01; compared with group K: ^Δ^*P *< 0.05, ^ΔΔ^*P *< 0.01; compared with group C: ^□^*P *< 0.05, ^□□^*P *< 0.01.

The survival rates in group S were higher than those in the ischemia-reperfusion groups, and the 3^rd ^day's survival rates in group M were lower than those in group K and C while they were higher than those in group CP (*P *< 0.05), there was no significant difference between group K and group C(*P *> 0.05). [3^rd ^day's survival rates: group S 92%(22/24), group M 42%(10/24), group K 75%(18/24), group C 75%(18/24), group CP 12.5%(3/24)] (Fig. 2)

### Changes of serum biochemical indicator in the survival rats

Compared with those on the 3^rd ^day in group S, the concentrations of TP and ALB in group M decreased significantly while S/L and LDH increased significantly (*P *< 0.05) (Table 1); the concentrations of TP and ALB in group K decreased significantly while S/L, CK, and LDH increased significantly(*P *< 0.05) (Table 1); the concentrations of TP, ALB and GLB in group C decreased significantly while S/L and LDH increased significantly (*P *< 0.05) (Table 1); the concentrations of TP, ALB and GLB in group CP decreased significantly while AST, S/L and CK increased significantly (*P *< 0.05) (Table 1).

**Table 1:** changes of serum biochemical indicator (mean ± SD)

	n	AST (U/L)	ALT (U/L)	S/L	TP (g/L)	ALB (g/L)	GLB (g/L)	A/G	BUN (mmol/L)	CRE (μmmol/L)	LDH (U/L)	CK (U/L)
S	8	82 ± 21	56 ± 30	1.6 ± 0.4	71 ± 4	32 ± 3	38 ± 3	0.9 ± 0.1	6.0 ± 1.1	33 ± 3	69 ± 23	126 ± 29
M	8	121 ± 27	37 ± 7	3.4 ± 1.4**	60 ± 9*	24 ± 2**	36 ± 8	0.7 ± 0.1	6.0 ± 0.9	25 ± 3	235 ± 92*	205 ± 104
K	8	118 ± 47	40 ± 12	2.9 ± 0.6*	59 ± 3*	24 ± 2**	35 ± 3	0.7 ± 0.1	5.1 ± 1.0	28 ± 4	160 ± 60*	338 ± 114**
C	8	114 ± 39	40 ± 12	2.9 ± 0.5*	55 ± 6**	24 ± 1**	31 ± 5*	0.8 ± 0.1	4.3 ± 0.9**^##^	28 ± 3	138 ± 34**	251 ± 147
CP	3	301 ± 91**^##ΔΔ□□^	45 ± 19	4.6 ± 0.9**^Δ^	50 ± 12**	22 ± 5**	28 ± 8*	0.8 ± 0.1	5.3 ± 0.7	34 ± 6^#^	343 ± 61	41 ± 149**

Compared with group S:*P < 0.05, **P < 0.01; compared with group M: ^#^*P *< 0.05, ^##^*P *< 0.01; compared with group K: ^Δ^*P *< 0.05, ^ΔΔ^*P *< 0.01; compared with group C: ^□^*P *< 0.05, ^□□^*P *< 0.01.

The concentration of AST in group CP was higher than that in group M, K, and C(*P *< 0.05) (Table 1); and the concentration of CRE in group CP was higher than that in group M (*P *< 0.05) (Table 1).

There were no significant differences in group M, C and group K (*P *> 0.05). (Table 1)

### Changes of levels of TNF-α, IL-1β, IL-6 and IL-10 in intestine in the survival rats

Compared with group S in the 3^rd ^day, the level of IL-10 in group M decreased significantly while IL-6 and TNF-α increased significantly (*P *< 0.05) (Table 2); the level of IL-10 in group C decreased significantly (*P *< 0.05) (Table 2). There were no significant differences in group K and CP compared with group S(*P *> 0.05). (Table 2)

**Table 2:** Changes of the levels of TNF-α, IL-1β, IL-6, IL-10, IMMC counts and Chiu's score in the intestine(mean ± SD)

	n	IL-10 (pg/mg)	IL-1β (pg/mg)	IL-6 (pg/mg)	TNF-α (pg/mg)	IMMC counts (n/field)	Chiu's score
S	8	33 ± 4	613 ± 194	6 ± 2	30 ± 11	24 ± 14	0.4 ± 0.5
M	8	21 ± 5**	657 ± 129	40 ± 16**	43 ± 5*	26 ± 11	1.0 ± 0.5
K	8	27 ± 3	750 ± 150	13 ± 5^#^	27 ± 7^##^	28 ± 13	0.6 ± 0.6
C	8	22 ± 5**	678 ± 170	29 ± 12**^□^	25 ± 8^##^	31 ± 12	1.0 ± 0.5
CP	3	34 ± 3	814 ± 234	28 ± 5	33 ± 5	29 ± 7	1.0 ± 1.0

Compared with group S:*P < 0.05, **P < 0.01; compared with group M: ^#^*P *< 0.05, ^##^*P *< 0.01; compared with group K: ^Δ^*P *< 0.05, ΔΔ*P *< 0.01; compared with group C: ^□^*P *< 0.05, ^□□^*P *< 0.01.

Compared with group M, the levels of IL-6 and TNF-α in group K decreased significantly (*P *< 0.05). (Table 2)

There were no significant differences between group K and group C(*P *> 0.05). (Table 2)

### Changes of intestinal mucosa under light microscope and Chiu's score in the survival rats

**Figure 3:**
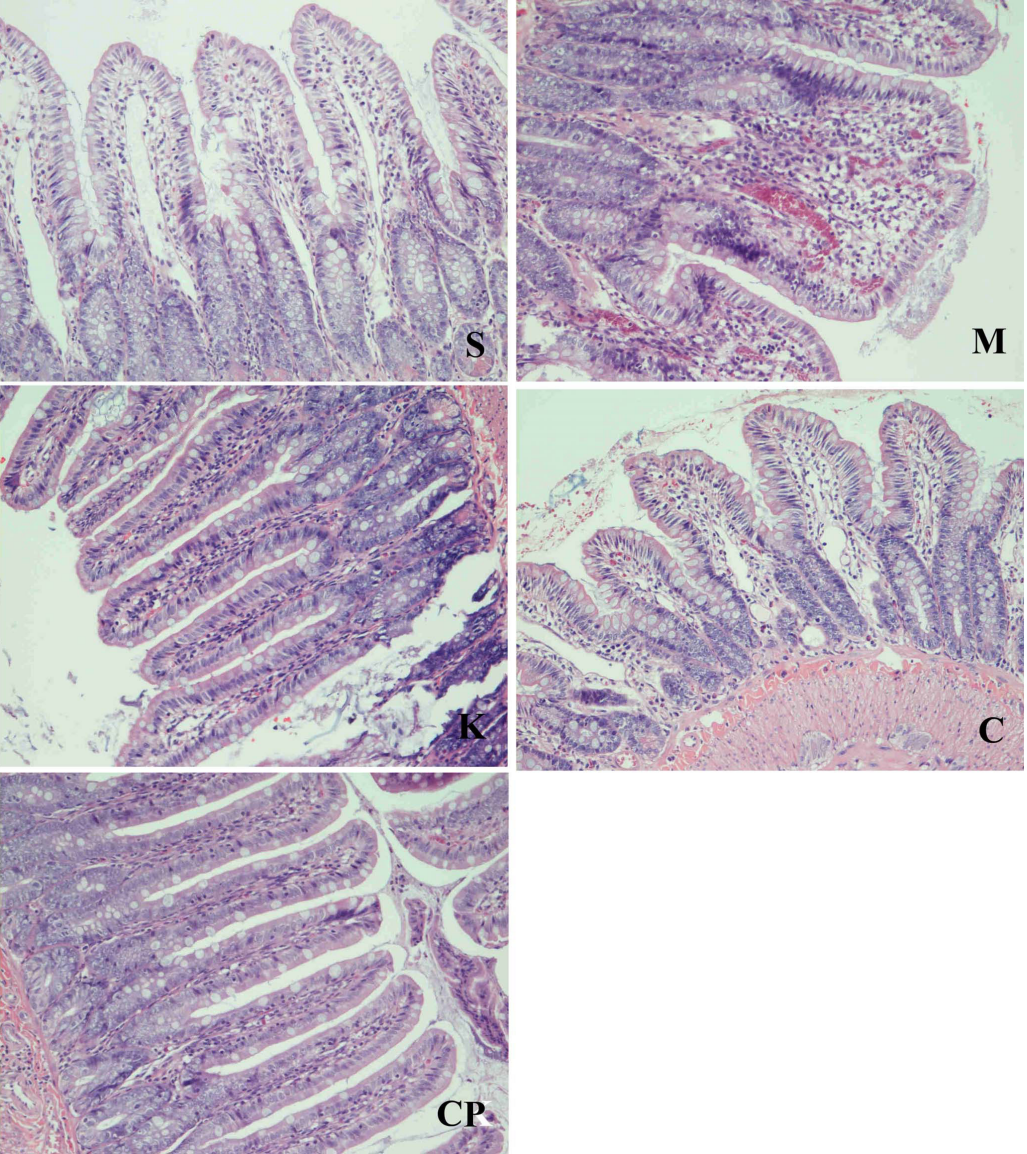
**Microscopic appearance after hematoxylin and eosin staining (× 200).** The villus and glands were normal and no inflammatory cell infiltration was observed in mucosal epithelial layer in sham group. Light edema of mucosa villus and infiltration of few necrotic epithelial inflammatory cells neutrophil leukomonocyte were found in mucosa epithelial layer in M, K, C and CP groups.

The villus and glands were normal. No inflammatory cell infiltration was observed in mucosal epithelial layer in sham group. Slight edema of mucosa villus and infiltration of few necrotic epithelial inflammatory cells neutrophil leukomonocyte were found in mucosa epithelial layer in M, K, C and CP groups. (Fig 3)

There were no significant differences in Chiu's score among the five groups on the 3^rd ^day (*P *> 0.05). (Table 2)

### Changes of counts of IMMC and ultrastructure in the survival rats

**Figure 4:**
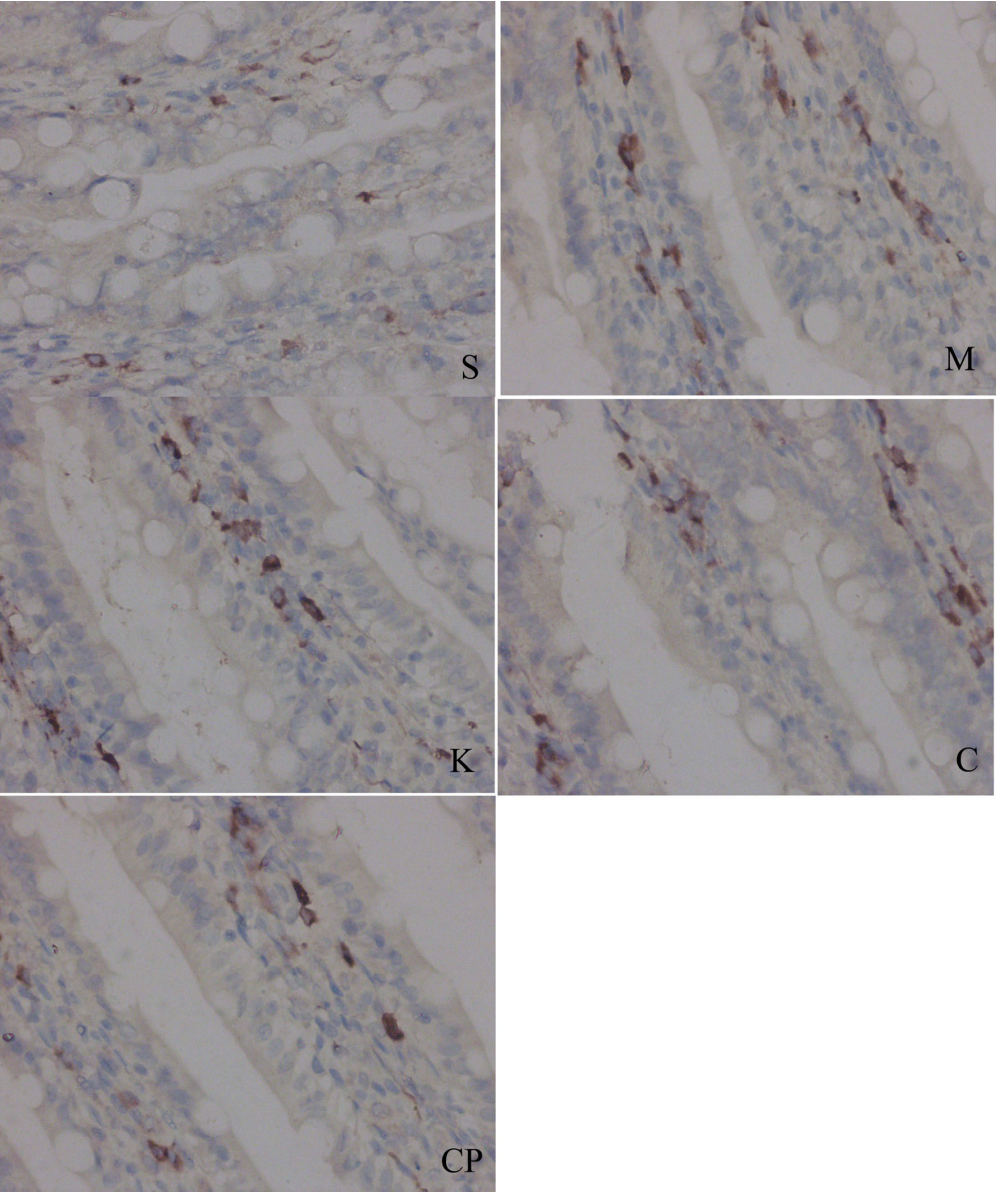
**Immunohistochemical detection of tryptase in small intestine of rats in each group (× 400).** Brownish granules in the cytoplasm were recognized as positive staining for tryptase.

**Figure 5:**
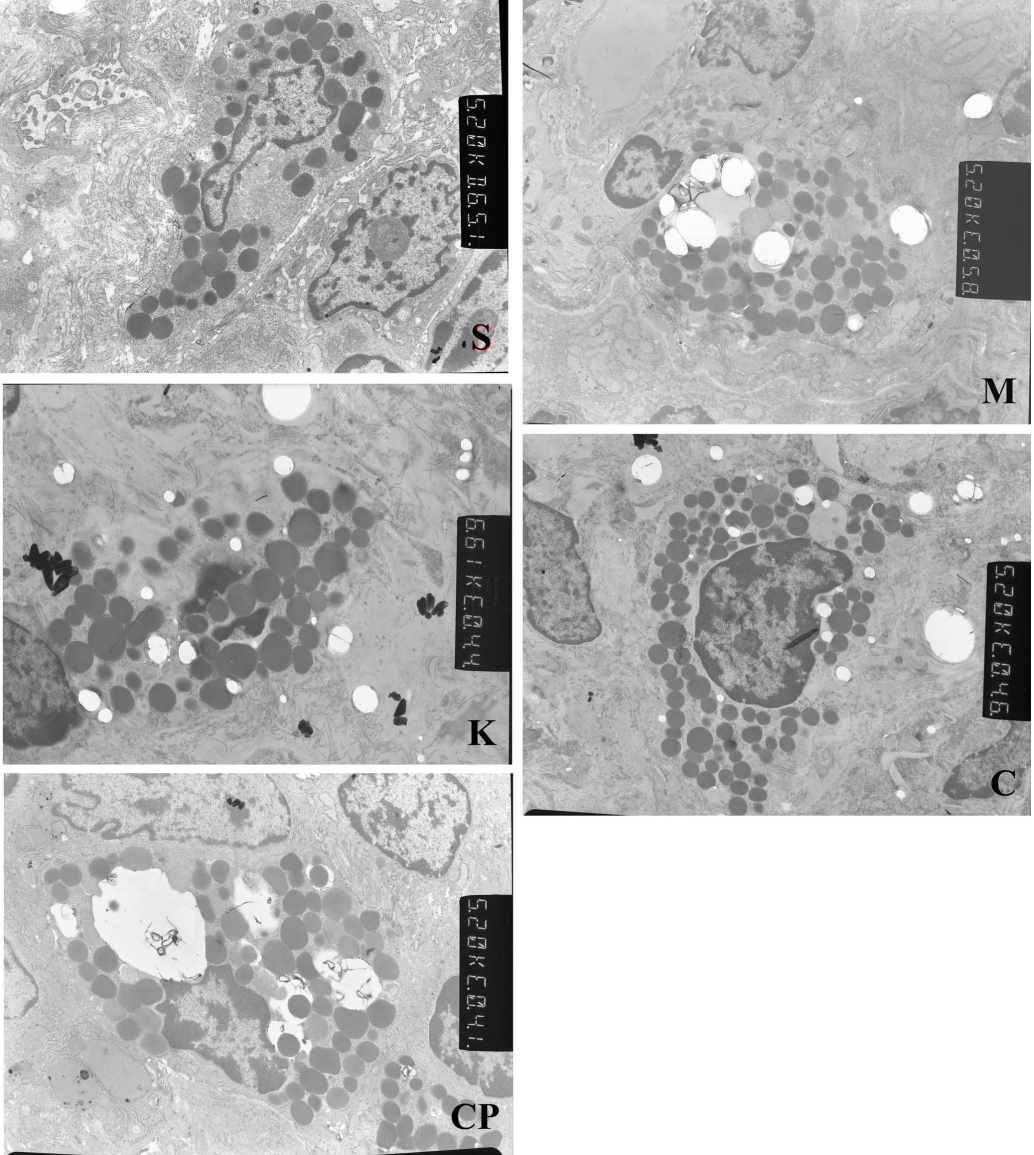
**Ultrastructure of intestinal mucosal mast cells of rats in each group (× 10,000).** There are abundant vacuolus with a reduction in granulation in their endochylema in group M and CP; Granulation in endochylema is obvious (Is this right?). There is no vacuolus in their endochylema in the sham group. These changes in ultrastructure are ameliorated by treatment with Cromolyn Sodium and kotifen in group C and K.

There were no significant difference in the number of IMMC among the five groups in the survival rats(*P *> 0.05) (Fig. 4, Table 2). The ultrastructure of IMMC was normal in the sham group. There were abundant vacuoles with reduced number of granules in their endochylema in groups M and CP. There were fewer swollen granules in IMMC homogeneity in groups C and K (Fig. 5).

### Changes of lung histology in the survival rats

**Figure 6:**
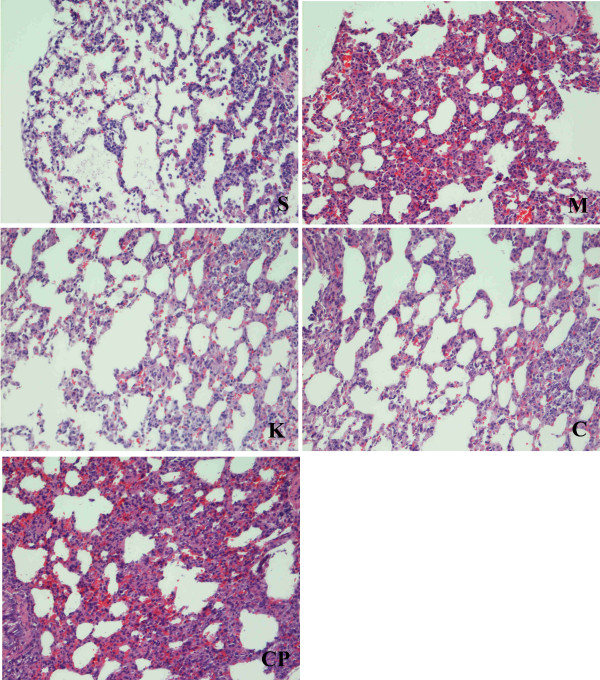
**Histological structure of lung of rats in each group (HE Staining, × 200).** The lung histological structure was normal in sham-operated group, while the lung tissues were obviously damaged with edema, hemorrhage, and inflammatory cell infiltration in group M and CP, and the injury was ameliorated in group C and K.

The lung histological structure was normal in group S, while the lung tissues were obviously damaged with edema, hemorrhage, and inflammatory cell infiltration in groups M and CP, and the injury was ameliorated in groups C and K. (Fig 6)

## Discussion

IIRI is a significant problem in small bowel transplantation, abdominal aortic aneurysm surgery, cardiopulmonary bypass, strangulated hernias, and neonatal necrotizing enterocolitis [10]. Most reports about IIRI selected rats as the animal models. Differences in intestinal ischemia time would result in different survival rates after ischemia reperfusion. The ideal survival rate in intestinal ischemia-reperfusion injury animal model should be 40%~50% after seven days as those survival could provide an ideal model for treatment or study. Our study found that with the prolonged intestinal ischemia time, the survival rate after ischemia reperfusion decreased significantly. The survival rates after intestinal ischemia for 75 min were about 40%~50% during the 1^st ^to 7^th ^day after reperfusion in Sprague-Dawley rats. In addition, we observed most animals died on the 1^st ^day after reperfusion, and there were almost no animals died on the 4^th ^to 7^th ^day after reperfusion. As a result, the ischemia time of 75 min and 3 days' reperfusion is appropriate in our study.

IMMC is an enriched source of inflammatory mediators such as histamine, prostaglandin D2, leukotriene, IL-3, IL-4, IL-5, IL-6, IL-8, IL-10, IL-13, IL-16, TNF-α, and more [11]. Boros and his lab reported that mucosal mast cell de-granulation plays an important role in the initiation of tissue injury after intestinal ischemia-reperfusion injury, and depletion of mast cells with compound 48/80 pretreatment prior to ischemia decreased the severity of mucosal damage [6]. Kalia found the survival rate increased significantly after ketotifen pretreatment [8]. Andoh A reported that intestinal ischemia-reperfusion treatment induce a marked increase in mucosal permeability and IMMC degranulation, while the mucosal permeability and IMMC degranulation are significantly attenuated in mast cell deficient Ws/Ws rats [5]. Our previous study also proved that IIRI induces IMMC degranulated and the histamine concentration in intestine decreased [12], administrated cromolyn sodium could attenuate intestinal damage caused by IIRI [13]. The above studies suggest that stabilization of mast cells or degranulation of mast cells prior to ischemia may be a key mechanism to protect the gastrointestinal tract from injury. However, the studies about the effects of stabilization or de-granulation of mast cells after ischemia on IIRI and the survival rate have not previously been well investigated.

Cromolyn Sodium (CS) is a mast cell membrane stabilizer, it can inhibit the mast cell de-granulation and releasing of histamine, TNF-α, and other inflammatory mediators [14]. Ketotifen is a second generation histamine H2 blocker which has been used in the management of allergic disorders. In addition to histamine receptor antagonism, ketotifen has been found to inhibit the release of mast cell and neutrophil-derived proinflammatory mediators. Compound 48/80 is a condensation product of p-metoxyphenethyl-methylamine and formaldehyde, and is a potent inducer of mast cell de-granulation in rats [15]. The dosage of the above drugs were selected based on previous reports [8,16,17]. We found the survival rates during the 1^st ^to the 3^rd ^day with CS and ketotifen treatment were increased significantly compared with group M, while it decreased significantly with Compound 48/80 treatment compared with group M. The results indicate that stabilization of mast cells or antihistaminic after ischemia can also increase the survival rates while de-granulation of mast cells after ischemia can decrease the survival rates.

We observed functional changes in multi-organs in the survival rats, and analyzed the reasons that caused the rats to die as the dead animals couldn't be analyzed. Reperfusion with oxygenated blood after sustained ischemia is necessary to recover normal tissue and organ function. Intestinal mucosa is one of the best recovered organs in 24 hours after IIRI [18]. We also found there were no significant differences in Chiu's score among the five groups on the 3^rd ^day in the survival rats. The results also demonstrated that intestinal mucosa can be easily recovered from IIRI, which may the main reason that explains why the mortality didn't increase after the 3^rd ^day.

Surprisingly, we found that IMMC counts were the same in the survival rats in the five groups. Although there were abundant vacuoles with reduced numbers of granules in their endochylema after treatment with compound 48/80, and there were fewer swollen granules in IMMC homogeneity after treatment with CS or ketotifen. The results prove that compound 48/80 induces IMMC degranulation while CS and ketotifen inhibited IMMC degranulation. The findings indicated that inhibited IMMC from de-granulation may increase the survival rates after IIRI.

Previous studies have shown the important role of TNF-α, IL-1β and IL-6 for reperfusion-induced tissue injury and lethality [19]. IL-10 has anti-inflammatory properties and reduces tissue inflammatory injury following ischemia and reperfusion injury [20]. Our study found that the level of IL-6 in intestine in group model increases significantly compared with group sham on the third day, while the level of IL-10 in intestine decreased significantly on the 3^rd ^day. Treatment with cromolyn sodium and ketotifen can decrease the levels of TNF-α and IL-6 in intestinal significantly compared with group model. The results indicates that CS and ketotifen can also decrease inflammation after IIRI, which may be another reason for the increased survival rates after treatment with CS and ketotifen.

ALT is synthesized in cytoplasm and AST is synthesized in mitochondria. The increased ratio of S/L represents the severity of liver cell injuries. The increased CK and LDH reflects damages in myocardial cells peculiarly. TP respond to nutritional conditions and liver anabolic state. Our study found that the increased ratios of S/L, LDH and CK, and the decreased levels of TP in groups M, C and group K compared to group S in the survival rats. And the concentrations of AST and CRE increased significantly in group CP compared with group M. The lung tissues were obviously damaged with edema, hemorrhage, and inflammatory cell infiltration in group M and CP, and the injuries were ameliorated after treatment with CS and ketotifen. The above results indicates that intestinal ischemia could not only induce intestinal reperfusion injury but also induced remote organ injury such as liver, heart and lung [21-23], and the remote organ injury may be one of the important reasons that cause the animal to die.

## Conclusion

Intestinal mucosal mast cells play an important role in the intestinal ischemia-reperfusion injury. Treatment with CS and ketotifen prior to reperfusion and following reperfusion could increase the survival rates on the 3^rd ^day, while treatment with compound 48/80 could decrease the survival rates. Inhibition of mast cells from de-granulation provides a new treatment strategy to protect multiple organ injury induced by intestinal ischemia reperfusion. (The first 3 days) after ischemia-reperfusion injury is the most important time period for treatment. However, inflammatory injury to the intestines and damages to remote organs 3 days after reperfusion still exist.

## Abbreviations

CS: Cromolyn Sdium; CP: Compound 48/80; AST: glutamic-oxaloacetic transaminase; ALT: glutamic pyruvic transaminase; S/L: the ratio of AST compare ALT; TP: total protein; ALB: albumin; GLB: globulin; A/G: the ratio of ALB compare GLB; CK: phosphocreatine kinase; LDH: lactate dehydrogenase; BUN: urea nitrogen; CRE: creatinine; IIRI: Intestinal ischemia-reperfusion injury; IMMC: Intestinal mucosal mast cells.

## Competing interests

The authors declare that they have no competing interests.

## Authors' contributions

H-ZQ participated in the conception and design of the study, and revised the manuscript. G-XL established the experimental setup, and drafted the manuscript. H-PJ and W-J carried out biochemical study and carried out pathological examination. S-N and G-WL collected and analyzed the data. All authors read and approved the final manuscript.

## Pre-publication history

The pre-publication history for this paper can be accessed here:


